# Molecular analysis of ABCA4 and CRB1 genes in a Spanish family segregating both Stargardt disease and autosomal recessive retinitis pigmentosa

**Published:** 2008-02-04

**Authors:** Rosa Riveiro-Alvarez, Elena Vallespin, Robert Wilke, Blanca Garcia-Sandoval, Diego Cantalapiedra, Jana Aguirre-Lamban, Almudena Avila-Fernandez, Ascension Gimenez, Maria-Jose Trujillo-Tiebas, Carmen Ayuso

**Affiliations:** 1 Genetics Department, Fundacion Jimenez Diaz-CIBERER; Madrid, Spain.; 2 Department for Pathophysiology of Vision and Neuro-Ophthalmology, University Eye Hospital of Tübingen; Tübingen, Germany.; 3 Ophthalmology Department, Fundacion Jimenez Diaz-CIBERER;Madrid, Spain.

## Abstract

**Purpose:**

Stargardt disease (STGD), characterized by central visual impairment, is the most common juvenile macular dystrophy. All recessively inherited cases are thought to be due to mutations in the *ABCA4* gene. Early-onset autosomal recessive retinitis pigmentosa (arRP) is a severe retinal degeneration that presents before the patient is ten years old. It has been associated with mutations in different genes, including *CRB1*. The aim of this study was to determine the genetic causes for two different retinal dystrophies, STGD and early-onset arRP, both segregating in one Spanish family.

**Methods:**

Mutational analyses were performed using the ABCR400 and Leber congenital amaurosis (LCA) genotyping microarrays. Additional scanning for mutations was conducted by denaturing high performance liquid chromatography (dHPLC); results were confirmed by direct sequencing.

**Results:**

A patient, who exhibited a STGD phenotype, was found to be homozygous for the p.Asn1805Asp (c.5413A>G) mutation in *ABCA4*. However, his affected sister, who had the arRP phenotype, was found to be heterozygous for this allele; no other sequence change could be found in *ABCA4.* Analysis using the LCA chip revealed the p.Cys948Tyr mutation in *CRB1* in heterozygous state. A second mutation (p.Trp822ter) was found in the *CRB1* gene in the affected female by denaturing high performance liquid chromatography (dHPLC) and direct sequencing.

**Conclusions:**

Two distinct retinal dystrophies with mutations affecting two different genes cosegregated in this family. The presence of two different phenotypes associated with mutations in two distinct genes in one single family must be considered especially when dealing with retinal dystrophies which bear high carrier frequencies in general population.

## Introduction

Stargardt disease (STGD1, MIM #248200) is the most common hereditary macular dystrophy affecting children, with a prevalence of approximately 1:10000 [1]. It is characterized by central visual loss, atrophy of the retinal pigment epithelium (RPE) that resembles a “beaten-bronze appearance,” and the distribution of orange yellow flecks around the macula and/or midperiphery of the retina [2]. STGD is predominantly inherited as an autosomal recessive trait, although an autosomal dominant form has been also described [3]. Biallelic mutations in *ABCA4* are found in most patients with autosomal recessive STGD (arSTGD) [4] as well as in some patients with autosomal recessive retinitis pigmentosa (arRP) [5] and autosomal recessive cone-rod dystrophy (arCRD) [6]. Heterozygote carrier frequency is particularly high in the general population [7].

RP with paraarteriolar preservation of RPE (RP12, MIM#600105) is a specific form of RP characterized by preserved paraarteriolar RPE (PPRPE) in the early-to-middle stages of disease. Patients experience night blindness and develop a progressive loss of visual fields before they are 10 years old. By the time they are 20, patients have severe visual impairment because of early macular involvement. Other features reported with this type of RP are hyperopia, nystagmus, optic-nerve–head drusen, vascular sheathing, and maculopathy [8,9]. Mutations in the *CRB1* (*Crumbs homolog 1*) gene have been found in patients with RP12, with and without PPRPE, RP patients who developed Coats-like exudative vasculopathy, and in 10%–13% of patients with Leber congenital amarousis (LCA). This gene maps to chromosome 1q31.3 and contains 12 exons. RP caused by *CRB1* mutations is inherited in an autosomal recessive manner [10-12].

In this study, we used the ABCR400 and LCA microarrays to investigate the disease-causing mutations in one family who presented with two distinct phenotypes: STDG and early onset RP with a PPRPE phenotype.

## Methods

### Ascertainment of patients

This molecular study was reviewed and approved by the Ethics Committee of Fundacion Jimenez Diaz, and it was performed according to the tenets of the Declaration of Helsinki and further reviews (Edinburgh, 2000; www.wma.net). A total number of 129 Spanish families affected with different retinal dystrophies due to mutations in the *ABCA4* gene were studied. Of these, we selected one family segregating both STGD and early-onset arRP for further investigation.

### Clinical Evaluation

Thorough clinical ophthalmic and electrophysiological examinations were performed in both affected patients, which included a comprehensive ophthalmological and family history, funduscopic examination after pupillary dilation, static perimetry, best corrected visual acuity exam, and color vision testing. For the STGD patient, fluorescein angiography was also performed. Electrophysiological assessment included full-field electroretinogram (ERG), according to standards of the International Society for Clinical Electrophysiology of Vision [13,14].

### Molecular methods

#### DNA extraction

Peripheral blood samples with ethylenediaminetetraacetic acid (EDTA) anticoagulant were collected from each member of the family. Genomic DNA was extracted using an automated DNA extractor (BioRobot EZ1, QIAGEN, Hilden, Germany). Prior to use, DNA samples were preserved frozen.

### Genotyping microarray

STGD patients were analyzed for variants on the ABCR400 microarray (www.asperbio.com), as described elsewhere [7]. The 50 exons of the *ABCA4* gene, including the intron-exon junctions, were amplified by PCR primers previously described [15] to confirm the results obtained from the microarray.

LCA and early-onset samples were screened on the LCA genotyping microarray (www.asperbio.com), as previously reported [16]. The 12 exons of the *CRB1* gene, including the intron-exon boundaries, were amplified by PCR and sequenced with primers previously described [17] to test the results.

### Direct sequencing

Sequencing reactions were performed using the 4 dye terminator cycle sequencing ready reaction kit (dRhodamine DNA Sequencing Kit; Applied Biosystems, Foster City, California). Sequence products were purified through fine columns (Sephadex G-501; Princetown Separations, Adelphia, New Jersey) and resolved in an ABI Prism 3100 (Applied Biosystems).

### Denaturing high-performance liquid chromatography

Denaturing high-performance liquid chromatography (dHPLC) sample screening was performed on a DNA fragment analysis system (WAVE; Transgenomic, Omaha, Nebraska). The PCR products (5 μl) were loaded on a C_18_ reversed-phase column (DnaSep column; Transgenomic). Hetero- and homodimer analysis was performed with an acetonitrile gradient formed by mixing buffers A and B (WAVE Optimized, Transgenomic). The flow rate was 0.9 mL/min, and DNA was detected at 260 nm. For each DNA region, dHPLC conditions were established by a triple analysis 1 °C to 3 °C above and below the mean melting temperature predicted by software simulation.

Because dHPLC usually does not differentiate between the wild-type and the homozygous mutant sample, all unknown samples were mixed in a 3:1 proportion with a control sample at the end of each PCR session. Before dHPLC analysis, heteroduplexes were formed by denaturing the PCR product at 95 °C for 5 min and cooling it to room temperature.

### Haplotype analysis

Haplotypes were generated using three microsatellite markers flanking the *ABCA4* gene (TEL-*D1S435*-*D1S2804*-*ABCA4*-*D1S236*-CEN) and four microsatellite markers flanking the *CRB1* gene (TEL-D1S1660-*CRB1*-D1S2757-D1S2816-D1S408-CEN). After amplification by PCR, fluorescent-labeled products were mixed and electrophoresed on the ABI Prism 3100 (Applied Biosystems). For haplotype reconstruction, we used an informatic program (Cyrillic version 2.1; www.cyrillicsoftware.com).

## Results

### Clinical findings

**Figure 1:**
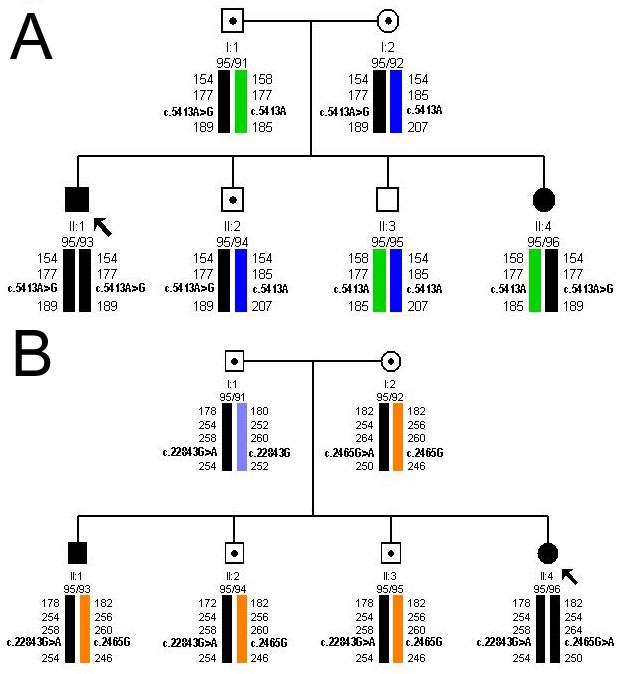
Pedigrees from a Spanish family cosegregating Stargardt disease and early-onset retinitis pigmentosa. **A**: Haplotype analysis showing microsatellite markers flanking the *ABCA4* gene (TEL-*D1S435*-*D1S2804*-*ABCA4*-*D1S236*-CEN) confirmed the Stargardt disease phenotype in II:1. His affected sister (II:4) was found to be a carrier of one disease-associated allele. **B**: Haplotype analysis with markers flanking the *CRB1* gene (TEL-D1S1660-*CRB1*- D1S2757- D1S2816-D1S408-CEN) showed cosegregation of the disease. Three brothers (II:1, II:2, II:3) were found to be carriers of the p.Cys948Tyr allele.

**Figure 2:**
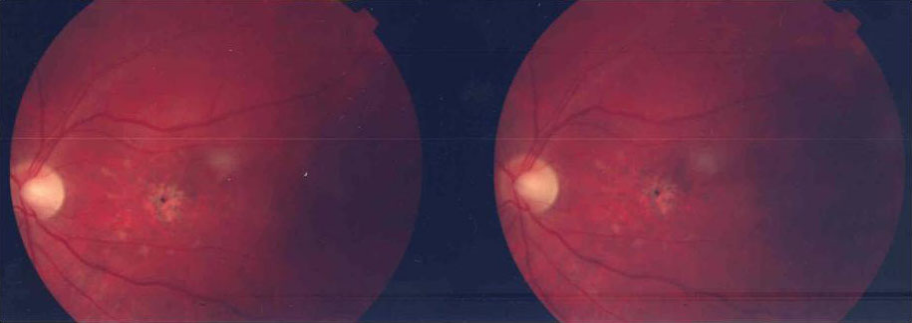
Funduscopic photograph of a patient with Stargardt disease. Funduscopy revealed a maculopathy with retinal pigment epithelium atrophy and hyperpigmentation and few central yellowish flecks. A slight temporal papillary pallor was present and retinal vessels showed no constriction.

**Figure 3:**
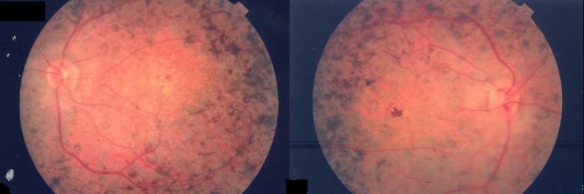
Funduscopic photograph of a patient with autosomal recessive retinitis pigmentosa. Funduscopy showed pigment spots homogeneously scattered through the retina with preservation of the paraarteriolar retinal pigmentary epithelium.

From our cohort of patients we selected one family (Figures 1A and 1B) with two distinct phenotypes (STGD and early-onset RP with PPRPE) for further investigation. The 33-year-old STGD patient initially presented with visual acuity loss, photophobia, myopia, and astigmatism starting at the age of 14 years (Figure 1A, individual II:1). At that time, no restriction of visual fields or night vision disturbances were noticed. At age 26, he had a visual acuity of 10/100 in both eyes on clinical examination; anterior segments were normal. Anterior segments were normal, except for a slight subcapsular opacification of the posterior pole of the lens. Funduscopy revealed a maculopathy with RPE atrophy and hyperpigmentation and a few central yellowish flecks; the peripheral retina showed no changes in comparison to exam performed when he was 14 years. A slight temporal papillary pallor was present, and retinal vessels showed no constriction (Figure 2). Static perimetry could not reveal central scotomas, but shallow relative peripheral scotomas in both eyes. The full-field ERG response showed slightly reduced—but still within the normal range—amplitudes for rod, mixed cone-rod, cone single flash, and cone flicker, respectively. His 16-year-old sister presented a different retinal phenotype, resembling early-onset RP. When she was two years old, her parents noticed a deficit of the peripheral visual field. At the age of 14, she developed night vision deficits and reduction of central visual acuity. Ophthalmic evaluation of the patient at that time documented reduced visual acuity (10/100 right eye, 20/100 left eye) and diminished visual fields, hyperopia, astigmatism, and nystagmus. Anterior segments were normal. Funduscopy revealed roundish pigments distributed across the entire retina including peripheral retina, posterior pole, and macular region. The retinal vessels showed filliform constriction, and the optic disc was normal. Visual fields were concentrically constricted with small remaining central and nasal islands (less than 10 degrees). ERG responses (photopic and scotopic) were not discernible from noise anymore. The last fundus examination was performed when she was 26 years old. It showed pigment spots homogeneously scattered through the retina with preservation of the PPRPE (Figure 3).

### Molecular analyses

This family has been previously analyzed [18,19]. In this molecular study, we described a 33-year-old man diagnosed with STGD (II:1), who was homozygous for the missense *ABCA4* p.Asn1805Asp (c.5413A>G) mutation. No other pathogenic mutation was found in the screening of the gene using the ABCR400 microarray. Four other members of the family, both parents and two siblings of the proband, were heterozygous for the *ABCA4* c.5413A>G allele. While the parents and the brother were unaffected, the sister presented with a severe early onset arRP, which could not be explained by the *ABCA4* genotype. Although she was tested for novel *ABCA4* mutations by dHPLC, no additional variants were found. Haplotype analyses confirmed the segregation of the disease-associated allele in this family (Figure 1A).

Following these molecular analyses, the affected sister with the early-onset RP phenotype was tested for mutations by screening on the LCA microarray. She was found to be heterozygous for the *CRB1* p.Cys948Tyr (c.2843G>A) mutation. Her three brothers also carried this change. Haplotype analyses showed cosegregation within the family (Figure 1B). Following, the 12 exons of the *CRB1* gene were screened for variants by dHPLC, where DNA fragment corresponding to exon 7 showed abnormal chromatographic pattern. Sequence analysis of the fragment led to the identification of the novel p.Trp822ter (c.2465G>A) mutation, which was also present in her healthy mother.

## Discussion

Retinal dystrophies are genetically and clinically heterogeneous. Through the course of a conventional screening performed on Spanish families with retinal dystrophies, we identified one family with two siblings affected by STGD and early-onset RP, respectively. Molecular analyses revealed that mutations in two different genes were implicated: *ABCA4* and *CRB1*. The STGD patient carried the homozygous p.Asn1805Asp allele in the *ABCA4* gene, while the RP patient had the disease-associated alleles p.Cys948Tyr and p.Trp822ter cosegregating in the *CRB1* gene.

The p.Asn1805Asp allele may act as a modifying factor for the RP phenotype in the female patient. Similarly, a possible modifier effect of the mutant *CRB1* allele for the STGD patient could be involved.

In previous analyses performed in this family, homozygosity was tested by haplotype construction with markers close to several RP genes, due to suspected consanguinity. In no case was the affected sister found to be homozygous for any of these screened regions [19]. Our haplotype analysis neither revealed homozygosity for markers close to *ABCA4* and *CRB1* genes.

Several families with both STGD and arRP have been reported and associated to different *ABCA4* mutations [19-23]. To the best of our knowledge, this report is the first description of two different genes implicated in two distinct autosomal recessive retinal dystrophies in one family. To date, there has been a description of one consanguineous family segregating mutations in two different genes, both causing Bardet Biedl Syndrome [24].

Thompson and colleagues suggested that the total number of recessive mutations per chromosome in any one individual may be low [25]. However, the family in the present study is segregating two autosomal recessive ophthalmologic diseases, with mutations in two different genes, both located at chromosome 1.

The present study was part of an analysis performed on 129 Spanish families segregating different retinal dystrophies due to mutations in *ABCA4. *In this work, a total of 18 patients were diagnosed of arRP. Nevertheless, we did not identify any patient harbouring both disease-associated alleles. Therefore, we could not establish a RP phenotype caused by mutations in this particular gene [26]. Similarly, Valverde et al. recently observed that *ABCA4* disease-associated variants are implicated in STGD and CRD phenotypes, but their implication in RP still remains unclear. However, a modulating effect could be suspected [27].

The relative large sizes of these genes, containing 50 and 12 exons respectively, make the molecular scanning of *ABCA4* and *CRB1* particularly labor-intensive. The ABCR400 and LCA chips have become reliable and rapid mutation detection tools. However, because of the high frequency of rare mutations reported in these genes, additional mutational scanning should be performed by dHPLC. This technique is especially applicable for those *ABCA4*/*CRB1* patients for whom chips have found one or no mutations.

In this family, two distinct retinal dystrophies cosegregated with mutations in two different genes. An accurate clinical diagnosis obtained from comprehensive ophthalmologic examination provides the basis for conducting the molecular analysis of one particular gene. The identification of the causative mutation was helpful for confirming diagnosis and providing genetic counseling and possibly predicting the further course of the disease. The presence of two different phenotypes associated with mutations in two distinct genes in one single family must be considered especially when dealing with retinal dystrophies that bear high carrier frequencies in general population.
